# Investigating the spatial resolution of EMG and MMG based on a systemic multi-scale model

**DOI:** 10.1007/s10237-022-01572-7

**Published:** 2022-04-20

**Authors:** Thomas Klotz, Leonardo Gizzi, Oliver Röhrle

**Affiliations:** 1 Institute for Modelling and Simulation of Biomechanical Systems, Pfaffenwaldring 5a, 70569 Stuttgart, Germany; 2 Stuttgart Centre for Simulation Science (SimTech), Pfaffenwaldring 5a, 70569 Stuttgart, Germany

**Keywords:** Neuromuscular physiology, Skeletal muscle, Biosignal, Electromyography, Magnetomyography, Continuum model

## Abstract

While electromyography (EMG) and magnetomyography (MMG) are both methods to measure the electrical activity of skeletal muscles, no systematic comparison between both signals exists. Within this work, we propose a novel in silico model for EMG and MMG and test the hypothesis that MMG surpasses EMG in terms of spatial selectivity, i.e. the ability to distinguish spatially shifted sources. The results show that MMG provides a slightly better spatial selectivity than EMG when recorded directly on the muscle surface. However, there is a remarkable difference in spatial selectivity for non-invasive surface measurements. The spatial selectivity of the MMG components aligned with the muscle fibres and normal to the body surface outperforms the spatial selectivity of surface EMG. Particularly, for the MMG’s normal-to-the-surface component the influence of subcutaneous fat is minimal. Further, for the first time, we analyse the contribution of different structural components, i.e. muscle fibres from different motor units and the extracellular space, to the measurable biomagnetic field. Notably, the simulations show that for the normal-to-the-surface MMG component, the contribution from volume currents in the extracellular space and in surrounding inactive tissues, is negligible. Further, our model predicts a surprisingly high contribution of the passive muscle fibres to the observable magnetic field.

## Introduction

Movement relies on the complex interplay of the neural and musculoskeletal system. In short, the neuromuscular system comprises motor units, consisting of a motor neuron and all muscle fibres it innervates (Heckman and Enoka (2012)). Motor neurons integrate signals from the brain, sensory organs and recurrent pathways. Once a motor neuron surpasses its depolarisation threshold, it triggers an action potential that propagates along the respective axon to the neuromuscular junctions. The latter opens ion channels in the sarcolemma, i.e. the muscle fibre membrane, yielding an action potential that travels along the muscle fibre triggering an intracellular signalling cascade that ultimately leads to force production, cf., e.g. MacIntosh et al. (2006) and Röhrle et al. (2019).

From a physical point of view, an action potential represents a coordinated change of a membrane’s polarity and thus causes both a time-dependent electric field, i.e. due to the distribution of charges, and a magnetic field, i.e. due to the flux of charges. This can be exploited for observing a skeletal muscle’s activity via electromyography (EMG) or magnetomyography (MMG). Both signals contain information on the neural drive to the muscle and the state of the muscle, and, thus, can be both utilised to investigate various aspects of neuromuscular physiology. In the past, however, it was almost only EMG that has been used to study neuromuscular physiology (for an overview see Merletti and Farina (2016)). While EMG can be recorded either intramuscularly or from the body surface, from a practical point of view, non-invasive measurements are desirable. Signals obtained from surface EMG, however, exhibit a reduced spatial selectivity, as the volume conductive properties of subcutaneous tissues act as low-pass filter. This means that spatially distinct sources show more similar projections on the same EMG channel. Consequently, the separation and the accurate reconstruction of the bioelectric sources become challenging, if not unfeasible. As the magnetic permeability of biological tissues is close to the magnetic permeability in free space, the magnetic field generated by a skeletal muscle is not distorted by subcutaneous tissues (Malmivuo and Plonsey 1995; Oschman 2002). Hence, MMG has the potential to overcome the physical limitations of surface EMG. Further, in contrast to EMG, MMG recordings do not rely on sensor-tissue contacts and thus are particularly appealing for long term measurements, e.g. prosthesis control via implanted sensors Zuo et al. (2020). Although MMG was already first described by Givler in 1972, there still exist several challenges that limit its practical use. Most importantly the amplitude of the magnetic field induced by skeletal muscles is very low, i.e. in the range of picotesla to femtotesla and, thus, significantly lower than the earth’s magnetic field. This yields high technical demands for MMG recording systems (Zuo et al. (2020)), for example, with respect to the sensitivity, the detection range, the sampling rate, the shielding from magnetic noise, the size and portability of the sensor device as well as the cost of such recordings. Nevertheless, a few proof-of-concept studies, e.g. Reincke (1993), Broser et al. (2018), Llinás et al. (2020) and Broser et al. (2021), illustrate its feasibility for biomedical applications.

Despite originating from the same phenomenon, there hardly exist any studies that investigate the biophysical factors affecting MMG or compare MMG recordings with EMG. Beside experimental studies, systemic in silico models can be used to investigate the factors influencing bioelectromagnetic signals and to test hypothesis derived from experimental observations. Particularly, continuum field models have been successfully used for assisting the interpretation of EMG signals, e.g. Farina et al. (2002), Mesin (2005), Dimitrova et al. (1999), Lowery et al. (2002), Farina et al. (2004), Mesin et al. (2006), Mordhorst et al. (2015), Mordhorst et al. (2017) and Klotz et al. (2020). In contrast, models to simulate magnetic fields induced by skeletal muscles are still rare. Common to all MMG models is that they first calculate the current field, which is then used to obtain the magnetic field. For example, Broser et al. (2021) used a finite wire model to infer from their measurements the underling physiology. However, this approach could not explain some of their experimental observations. This is mainly due to the oversimplification of the muscle’s anatomy as well as its physiology. Zuo et al. (2020, 2021) followed a full-field approach, which was originally proposed by Woosley et al. (1985), to simulate the magnetic field of an isolated axon. Thereby, the muscle fibres and the extracellular connective tissue are modelled as spatially separated regions, whereby the coupling conditions are determined from a pre-computed transmembrane potential. While this approach allows to calculate both the electrical potential field and the magnetic field in a small tissue sample, the computational demands are substantially limiting its use for simulating larger tissue samples. Further, the decoupling of the transmembrane potential from the intracellular and extracellular potential fields is a simplification potentially limiting the credibility of the resulting modelling predictions.

To enable systematic in silico investigations for both EMG and MMG signals, we extend our homogenised multi-domain modelling framework (Klotz et al. (2020)) to predict both the electric field and magnetic field induced by skeletal muscles. After establishing the model, we first investigate the hypothesis that for non-invasive recordings MMG provides a better spatial selectivity than EMG, i.e. motivated by the fact that the magnetic permeability of biological tissues is approximately the same as in free space. Further, we use our model to quantify the contributions of different structural components to a muscle induced biomagnetic field.

## Methods

**Fig. 1:**
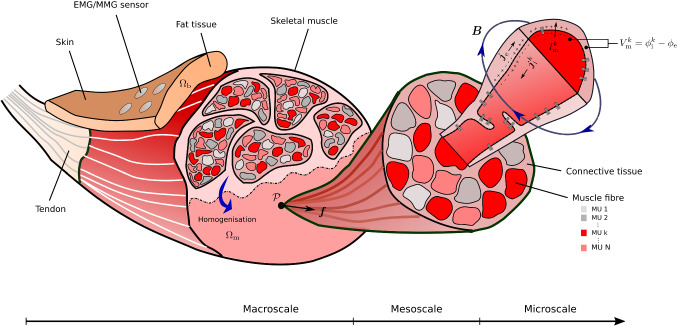
Schematic drawing illustrating the concept of the proposed multi-scale model. On the macroscale, the heterogeneous muscle structure is smeared and represented by an idealised and continuous multi-domain material. To couple the different domains, the multi-scale model still captures the most important features of the original structure. That is, the motor unit composition on the mesoscale and the interaction of one representative muscle fibre per motor unit with the extracellular space through the muscle fibre membrane on the microscale. Note that for the muscle fibres each colour represents a different motor unit. Based on those key properties, the continuous field approach predicts experimental measurable fields such as the transmembrane potentials $V_\mathrm {m}^k$, the extracellular potential $\phi _\mathrm {e}$ or the magnetic $\varvec{B}$ field. Non-invasive surface recordings, which are schematically illustrated by the sensors on the body surface, of the electrical potential field, i.e. via electromyography (EMG), or the magnetic field, i.e. via magnetomyography (MMG), are preferable as they yield minimal discomfort for a subject

### Modelling framework

This section presents the modelling framework for investigating EMG and MMG. The underlying governing equations are the quasi-static Maxwell’s equations as presented in Sect. 2.1.1. As the quasi-static approximation of Maxwell’s equations allows us to decouple the electric field from the magnetic field, we first derive a systemic multi-scale model to simulate the electro-physiological behaviour of skeletal muscles (cf. Sect. 2.1.2 and Klotz et al. (2020)) as well as electrically inactive tissue surounding the muscle (cf. Sect. 2.1.3). Based on the electric potential field in the body, the corresponding current densities, and, hence, the prediction of the magnetic field, can be calculated (cf. Sect. 2.1.4). Section 2.1.5 provides appropriate boundary conditions to guarantee existence and uniqueness for the solution of the derived system of partial differential equations.

#### Governing equations

In classical physics, the evolution of the electric field and the magnetic field is described by Maxwell’s equations. Since changes to the muscle induced electric and magnetic field are relatively slow, i.e. the characteristic time scale is in the range of milliseconds, the electrostatic and the magnetostatic approximation holds for modelling EMG and MMG. The differential form of the quasi-static Maxwell’s equations is given by, e.g. Griffiths (2013),

1a $$\begin{aligned} \text {div} \, \varvec{E} \ &= \ \frac{\rho }{\varepsilon _0} \ , \ (\text {Gauss's law}) \end{aligned}$$

1b $$\begin{aligned} \text {div} \, \varvec{B} \ &= \ 0 \ ,\ ({\text{Gauss's law for magnetism}}) \end{aligned}$$

1c $$\begin{aligned} \quad {\text {curl}}\, \varvec{E} \ &= \ \varvec{0} \ , \ ({\text{Faraday's law}}) \end{aligned}$$

1d $$\begin{aligned} \quad {\text {curl}}\, \varvec{B} \ &= \ \mu _0 \, \varvec{j} \ , \ ( {{\text{Amp}}{\grave {\text e}}{\text{re's}} \ {\text{law}}}) \end{aligned}$$

Therein, ${\text {div}}(\cdot )$ denotes the divergence operator, ${{\text {curl}}}(\cdot )$ denotes the curl operator, $\varvec{E}$ is the electric field, $\rho$ is the electric charge density, $\varepsilon _0$ is the vacuum permittivity, $\varvec{B}$ is the magnetic field (sometimes also referred to as magnetic flux density), $\mu _0$ is the vacuum permeability and $\varvec{j}$ is the total electric current density. Further, applying the div-curl identity to Ampère’s law (Eq. (1d)) yields the conservation of charges, i.e.

2 $$\begin{aligned} {\text {div}} \, \varvec{j} \ = \ 0 \ . \end{aligned}$$

Exploiting the fact that the electric field, $\varvec{E}$, is a conservative vector field and, thus, can be derived from a scalar potential, i.e. $\varvec{E} = - {\text {grad}} \, \phi$ with ${\text {grad}}(\cdot )$ being the gradient operator, reduces the number of state variables. Further, introducing the magnetic vector potential $\varvec{A}$ such that

3 $$\begin{aligned} \varvec{B} \ = \ {{\text {curl}}}\, \varvec{A} \end{aligned}$$

and calibrating it by Coulomb gauge, i.e. ${\text {div}} \, \varvec{A} = 0$, we obtain the quasi-static Maxwell’s equations in potential form:

4a $$\begin{aligned} {\text {div}}\big({\text {grad}} \, \phi \big) \ = \ - \frac{\rho }{\varepsilon _0} \ , \end{aligned}$$

4b $$\begin{aligned} {\text {div}}\big({\text {grad}}\, \varvec{A}\big) \ = \ - \mu _0 \, \varvec{j} \ . \end{aligned}$$

Next, we will introduce suitable modelling assumptions reflecting the electro-physiological properties of skeletal muscle tissue. Thereby note that for skeletal muscles, bound currents are assumed to be negligible and thus the total current density $\varvec{j}$ is equal to the ”free” current density (which is also sometimes called conductive current density).

#### Modelling the electrical behaviour of skeletal muscles

The electrical behaviour of skeletal muscles is simulated based on the multi-domain model presented in Klotz et al. (2020) and is briefly summarised here. Skeletal muscle tissue consists of muscle fibres associated with different motor units and extracellular connective tissue (cf. Fig. 1). The multi-domain model resolves this tissue heterogeneity by assuming that there coexist at each skeletal muscle material point $\mathcal {P}\in \mathrm {\Omega _m}$ an extracellular space and *N* intracellular spaces, with *N* denoting the number of motor units. Given this homogenised tissue representation, an electric potential is introduced for each domain, i.e. $\phi _\mathrm {e}$ and $\phi _\mathrm {i}^k$, $\forall \ k \in \mathscr {M}_\mathrm {MU}:=\{1,2,\ldots ,N \}$, where the subscripts $(\cdot )_\mathrm {e}$ and $(\cdot )_\mathrm {i}$ denote extracellular and intracellular quantities, respectively. Further, a transmembrane potential $V_\mathrm {m}^k$ is introduced for each motor unit, i.e.

5 $$\begin{aligned} V_\mathrm {m}^k \ = \ \phi _\mathrm {i}^k-\phi _\mathrm {e} \ , \ \forall \ k \in \mathscr {M}_\mathrm {MU} \ . \end{aligned}$$

The domains are electrically coupled, which is modelled by taking into account the most important features of skeletal muscles mesostructure and microstructure as well as the dynamics of the muscle fibre membranes. Thus, the multi-domain model can be classified as a multi-scale model.

The conservation of charges, i.e. Eq.(2), requires that all outward volume fluxes of the current densities from all domains are balanced at each skeletal muscle material point. For skeletal muscles it can be assumed that ions can only be exchanged between an intracellular domain and the extracellular space. There exist no current fluxes between the different intracellular domains. As the muscle fibres of the same motor unit are assumed to show similar biophysical properties, the coupling between an intracellular space *k* and the extracellular space is modelled by considering the interaction of one representative muscle fibre per motor unit with the extracellular space. Therefore, the current density outward volume flux of an intracellular domain is

6 $$\begin{aligned} {\text {div}} \, \varvec{j}_\mathrm {i}^k \ = \ A_\mathrm {m}^k I_\mathrm {m}^k \ , \ k \in \mathscr {M}_\mathrm {MU} \ , \quad \text {in} \ {\Omega _\mathrm{m}} \, , \end{aligned}$$

where $\varvec{j}_\mathrm {i}^k$ is the current density of motor unit *k* in a representative fibre-matrix cylinder. Further, $A_\mathrm {m}^k$ is the surface-to-volume ratio of a muscle fibre belonging to motor unit *k*, i.e. representing the geometry of the muscle fibres on the microscale, and $I_\mathrm {m}^k$ is the transmembrane current density, i.e. resolving the (microscale) behaviour of the muscle fibre membranes. The conservation of charges holds for each skeletal muscle material point if the current density outward volume flux from the extracellular domain is equal to the weighted sum of the transmembrane current densities, i.e.

7 $$\begin{aligned} {\text {div}} \, \varvec{j}_\mathrm {e} \ = \ - \sum _{k=1}^N \, f_\mathrm {r}^k A_\mathrm {m}^k I_\mathrm {m}^k \ , \quad \text {in} \ {\Omega _\mathrm{m}} \, , \end{aligned}$$

where $\varvec{j}_\mathrm {e}$ is the extracellular current density. Further, $f_\mathrm {r}^k$ is a (mesoscale) parameter, reflecting the motor unit composition at each skeletal muscle material point, i.e. the volume fraction of all muscle fibres belonging to motor unit *k* ($\forall k \ \in \mathscr {M}_\mathrm {MU}$) divided by the volume fraction of all muscle fibres.

The (conductive) current densities are related to the electric potential fields via Ohm’s law, i.e.

8 $$\begin{aligned} \begin{aligned} \varvec{j}_\mathrm {e}\ &= \ -\varvec{\sigma }_\mathrm {e} {\text {grad}} \, \phi _\mathrm {e} \ , \\ \varvec{j}_\mathrm {i}^k \ &= \ -\varvec{\sigma }_\mathrm {i}^k {\text {grad}} \, \phi _\mathrm {i}^k \ , \ \forall \ k \in \mathscr {M}_\mathrm {MU} \ , \end{aligned} \end{aligned}$$

where $\varvec{\sigma }_\mathrm {e}$ and $\varvec{\sigma }_\mathrm {i}^k$ denote the extracellular conductivity tensor and the intracellular conductivity tensors, respectively.

Finally, the transmembrane current densities, $I_\mathrm {m}^k$ ($\forall k \ \in \mathscr {M}_\mathrm {MU}$), are calculated from an electrical circuit model (Hodgkin and Huxley (1952); Keener and Sneyd (2009)) of the muscle fibre membranes via Kirchhoff’s current law, i.e.

9 $$\begin{aligned} \begin{aligned} I_\mathrm {m}^k \ = \ C_\mathrm {m}^k \dot{V}_\mathrm {m}^k \, + \, I_\mathrm {ion}^k(\varvec{y}^k,V_\mathrm {m}^k, I_{\mathrm {stim}}^k) \ , \\ \dot{\varvec{y}}^k \ = \ \varvec{g}^k(\varvec{y}^k,V_\mathrm {m}^k) \ , \\ \varvec{y}_0^k \ = \ \varvec{y}^k(t=0) \ , \\ V_\mathrm {m,0}^k \ = \ V_\mathrm {m}^k(t=0) \ . \\ \end{aligned} \end{aligned}$$

Therein, $C_\mathrm {m}^k$ is the membrane capacitance per unit area of a muscle fibre belonging to motor unit *k*, $I_\mathrm {ion}^k(\varvec{y}^k,V_\mathrm {m}^k, I_{\mathrm {stim}}^k)$ is the total ohmic current density through a representative membrane patch associated with MU *k* and $I_{\mathrm {stim}}^k$ is an external stimulus that is used to describe the motor nerve stimuli of motor unit *k* at the neuromuscular junctions. Further, $\varvec{y}^k$ is a vector of additional state variables, e.g. describing the probability of ion channels to be open or closed and $\varvec{g}^k(\varvec{y}^k,V_\mathrm {m}^k)$ is a vector-valued function representing the evolution equation for the membrane state vector $\varvec{y}^k$.

Combing Eqs. (5)–(9) yields for each $\mathcal {P}\in \mathrm {\Omega _m}$ the following system of coupled differential equations:

10a $$\begin{aligned} 0 \ &= \ {\text {div}} \left[ \varvec{\sigma }_\mathrm {e} {\text {grad}} \, \phi _\mathrm {e} \right] \, \nonumber \ \\ & \quad + \, \sum _{k=1}^N \, f_\mathrm {r}^k {\text {div}} \left[ \varvec{\sigma }_\mathrm {i}^k {\text {grad}} \left( V_\mathrm {m}^k + \phi _\mathrm {e} \right) \right] \ , \end{aligned}$$

10b $$\begin{aligned} & \displaystyle \frac{\partial V_\mathrm {m}^k}{\partial t} \ = \ \frac{1}{C_\mathrm {m}^k A_\mathrm {m}^k} \Big ( {\text {div}} \, \left[ \varvec{\sigma }_\mathrm {i}^k {\text {grad}} \left( V_\mathrm {m}^k + \phi _\mathrm {e} \right) \right]&\nonumber \hfill \\ & \quad \quad \quad \quad - \, A_\mathrm {m}^k I_\mathrm {ion}^k(\varvec{y}^k,V_\mathrm {m}^k, I_\mathrm{stim}^k) \Big ) \ , \ \forall \ k \in \mathscr {M}_\mathrm {MU} \ \end{aligned}$$

10c $$\begin{aligned} \dot{\varvec{y}}^k \ = \ \varvec{g}^k(\varvec{y}^k,V_\mathrm {m}^k) \ , \ \qquad \qquad \forall \ k \in \mathscr {M}_\mathrm {MU} \ . \end{aligned}$$

Further details can be found in Klotz et al. (2020).

#### Modelling the electrical behaviour of inactive tissues

Skeletal muscles are surrounded by electrically inactive tissues, e.g. connective tissues, fat or skin. Electrically inactive tissues have a strong influence on the electric potential on the body surface. From a modelling point of view, an inactive tissue is a volume conductor free of current sources, cf., e.g. Pullan et al. (2005), Mesin (2013) or Klotz et al. (2020). This yields a generalised Laplace equation for each material point within the body region $\mathcal {P}\in \mathrm {\Omega _b}$, i.e.

11 $$\begin{aligned} {\text {div}} \left[ \varvec{\sigma }_\mathrm {b} {\text {grad}} \, \phi _\mathrm {b} \right] \ = \ 0 \ , \quad \text {in} \ \mathrm {\Omega _b} \ , \end{aligned}$$

where $\phi _\mathrm {b}$ and $\varvec{\sigma }_\mathrm {b}$ are the body region’s electric potential and conductivity tensor, respectively.

#### Modelling magnetic fields induced by skeletal muscle’s electrical activity

Starting point for predicting the magnetic field is Ampère’s law, i.e. Eq. (1d) or (4b), which relates the magnetic field to the total current density. Exploiting that the radius of a muscle fibre is small compared to the characteristic length scale of the macroscopic continuum model and that the muscle fibres are (approximately) of cylindrical shape leads to the assumption that the contributions of the transmembrane currents to the macroscopic magnetic field cancel each other out. Further, assuming that for skeletal muscle tissue the magnetic susceptibility is approximately zero and that, within the limits of the quasi-static approximation, polarisation currents are negligible (cf. e.g., Malmivuo and Plonsey (1995)), then the overall current density is fully determined by the free (conductive) current densities. The latter is related to the electric potential field via Ohm’s law, and thus, for the homogenised multi-domain model the current density can be calculated for each domain independently (cf. Eq. (8)). To derive the right-hand side of Ampère’s law from the domain-specific current densities, we consider its integral form, i.e.

12 $$\begin{aligned} \oint _C \varvec{B} \, \mathrm {d}\varvec{l} \ = \ \mu _0 I_\mathrm {enc} \ . \end{aligned}$$

Therein *C* is an arbitrary closed curve, $I_\mathrm {enc}$ is the total current crossing *C* and $\mathrm {d}\varvec{l}$ is an infinitesimal line element. Equation 12 shows that the currents of the individual domains simply add up linearly. Since the current densities are given with respect to a representative fibre-matrix cylinder, the contributions of the intracellular current densities $\varvec{j}_\mathrm {i}^k$ ($\forall k \in \mathscr {M}_\mathrm {MU}$) need to be weighted by the (mesoscale) motor unit density factor $f_\mathrm {r}^k$. Accordingly, the magnetic vector potential for every material point within the muscle region $\mathcal {P} \in \mathrm {\Omega _m}$ is

13 $$\begin{aligned} & \begin{aligned} {\text {div}}\big({\text {grad}} \ \varvec{A}_\mathrm {m}\big) \ &=\ \mu _0 \, \Big ( \varvec{j}_\mathrm {e} \, + \, \sum _{k=1}^N f_\mathrm {r}^k \varvec{j}_\mathrm {i}^k \Big ) \ , \\ \Leftrightarrow {\text {div}}\big({\text {grad}} \, \varvec{A}_\mathrm {m}\big) \ &=\ - \mu _0 \, \Big ( \varvec{\sigma }_\mathrm {e} {\text {grad}} \, \phi _\mathrm {e} \\& \quad + \sum _{k=1}^N f_\mathrm {r}^k \varvec{\sigma }_\mathrm {i}^k {\text {grad}} \big(V_\mathrm {m}^k + \phi _\mathrm {e}\big) \Big ) \ . \end{aligned} \end{aligned}$$

Note, the potential formulation is chosen as this yields a Poisson-type equation for which various well-established numerical solution methods exist. Further, note that the linearity of the magnetostatic equations can be exploited to predict the contribution of each domain to the experimentally observable magnetic field. The body’s magnetic vector potential, $\varvec{A}_\mathrm {b}$, is calculated similarly:

14 $$\begin{aligned} \begin{aligned} {\text {div}}\big({\text {grad}} \, \varvec{A}_\mathrm {b}\big) \ &= \ \mu _0 \, \varvec{j}_\mathrm {b} \ , \\ \Leftrightarrow {\text {div}}\big({\text {grad}} \, \varvec{A}_\mathrm {b}\big) \ &= \ - \mu _0 \, \left[ \varvec{\sigma }_\mathrm {b} {\text {grad}} \, \phi _\mathrm {b} \right] \ , \end{aligned} \end{aligned}$$

where $\varvec{j}_\mathrm {b}$ is the current density in the body region. In contrast to the electric field equations, the magnetic field equations also need to consider the air surrounding the body. Since air can be assumed to be free of electric currents, it is modelled by

15 $$\begin{aligned} {\text {div}}\big({\text {grad}} \, \varvec{A}_\mathrm {f}\big) \ = \ \varvec{0} \ , \ \text {in} \ {\Omega _\text f} \ , \end{aligned}$$

where $\varvec{A}_\mathrm {f}$ is the magnetic vector potential within the surrounding space $\mathrm {\Omega _f}$.

Finally, the experimentally measurable magnetic field $\varvec{B}$ can be calculated straight forwardly from Eq.(3).

#### Boundary conditions

**Fig. 2:**
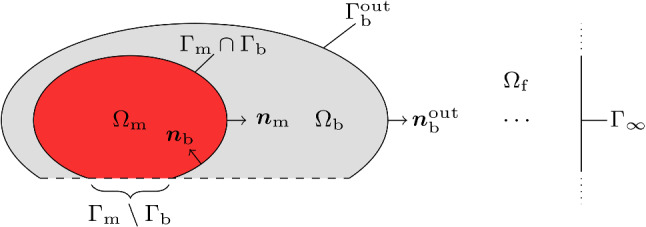
Schematic illustration of an arbitrary geometrical representation of muscle tissue $\mathrm {\Omega _m}$, the body region $\mathrm {\Omega _b}$, the surrounding space $\mathrm {\Omega _f}$, and its respective interfaces. Thereby, $\mathrm {\Gamma _m}$ denotes the muscle boundary with unit outward normal vector $\varvec{n}_\mathrm {m}$, $\mathrm {\Gamma _b^{out}}$ is the body surface with unit outward normal vector $\varvec{n}_\mathrm {b}^\mathrm {out}$ , $\mathrm {\Gamma _b}$ is an inner boundary of the body region with unit outward normal vector $\varvec{n}_\mathrm {b}$ and $\mathrm {\Gamma }_\infty$ refers to the set of infinitely distant points

Suitable boundary conditions are required to solve the partial differential equations presented in the previous sections. Recalling that muscle fibres are electrically insulated by their membranes, it is assumed that no charges can leave the intracellular domains at their boundary. This is modelled by applying zero Neumann boundary conditions to the intracellular potential, i.e.

16 $$\begin{aligned} \begin{aligned} \left[ \varvec{\sigma }_\mathrm {i}^k {\text {grad}} \, \phi _\mathrm {i}^k \right] \cdot \varvec{n}_\mathrm {m}& \ = \ 0 \ , \quad \ \ \ \, \text {on} \ \mathrm {\Gamma _m} \ , \\ \Leftrightarrow \left[ \varvec{\sigma }_\mathrm {i}^k {\text {grad}} \, V_\mathrm {m}^k \right] \cdot \varvec{n}_\mathrm {m} \\ = \ - \ \big [ \varvec{\sigma }_\mathrm {i}^k {\text {grad}}&\, \phi _\mathrm {e} \big ] \cdot \varvec{n}_\mathrm {m} \ , \ \ \ \text {on} \ \mathrm {\Gamma _m} \ , \end{aligned} \end{aligned}$$

where “$\cdot$” denotes the scalar product and $\varvec{n}_\mathrm {m}$ is a unit outward normal vector at the muscle surface $\mathrm {\Gamma _m}$ (cf. Fig. 2).

Further, it is assumed that no charges can leave the body, yielding zero Neumann boundary conditions for the electric potential in the body region, i.e.

17 $$\begin{aligned} \left[ \varvec{\sigma }_\mathrm {b} {\text {grad}} \, \phi _\mathrm {b} \right] \cdot \varvec{n}_\mathrm {b}^\mathrm {out} \ = \ 0 \ , \quad \text {on} \ \mathrm {\Gamma _b^{out}} \ . \end{aligned}$$

Therein, $\varvec{n}_\mathrm {b}^\mathrm {out}$ denotes a unit outward normal vector of the body surface $\mathrm {\Gamma _b^{out}}$ (cf. Fig. 2). In case that the outer surface of the simulated region is the skeletal muscle tissue’s boundary (or part thereof), the same assumption holds—however with zero Neumann boundary conditions for the extracellular potential, i.e.

18 $$\begin{aligned} \left[ \varvec{\sigma }_\mathrm {e} {\text {grad}} \, \phi _\mathrm {e} \right] \cdot \varvec{n}_\mathrm {m} \ = \ 0 \ , \quad \text {on} \ \mathrm {\Gamma _m} \setminus \mathrm {\Gamma _b} \ . \end{aligned}$$

While these are idealised cases typically not reflecting exact in vivo conditions, it should be noted that this boundary condition is still useful as most in silico experiments are restricted to a particular region of interest.

Finally, it is assumed that at the muscle–body interface, the extracellular potential $\phi _\mathrm {e}$, and the electric potential of the body region $\phi _\mathrm {b}$ are continuous, i. e.,

19 $$\phi _{e} \ = \ \phi _{b} \ ,\quad {\text{on}}\;\Gamma _{{\text{m}}} \cap \Gamma _{{\text{b}}}$$

Further, the current flux between the extracellular space and the body region is balanced, yielding

20 $$\begin{aligned} \big [ \varvec{\sigma }_\mathrm {e} {\text {grad}} \, \phi _\mathrm {e} \, - \, \varvec{\sigma }_\mathrm {b} {\text {grad}} \, \phi _\mathrm {b} \big ] \cdot \varvec{n}_\mathrm {m} \ = \ 0 \ , \ \text {on} \ \mathrm {\Gamma _m} \cap \mathrm {\Gamma _b} \ . \end{aligned}$$

Note electric potential fields are not unique, i.e. they can be shifted by an arbitrary scalar value. To make the solution unique, one can mimic/simulate a grounding electrode at a boundary location.

For the magnetic vector potential it can be assumed that far away from the muscle, i.e. the bioelectric sources, the magnetic field vanishes. Thus, zero Dirichlet boundary conditions are applied to all infinitely distant points $\mathrm {\Gamma }_\infty$, i.e.

21 $$\begin{aligned} \varvec{A} \ = \ \varvec{0} \ , \ \text {on} \ \mathrm {\Gamma }_\infty \ . \end{aligned}$$

It can be shown that the magnetic vector potential is continuous at the interface between two media (cf., e.g. Griffiths (2013)). This is modelled by

22a $$\begin{aligned} \varvec{A}_\mathrm {m} \ = \ \varvec{A}_\mathrm {b} \ , \ \text {on} \ \mathrm {\Gamma _m} \cap \mathrm {\Gamma _b} \ , \end{aligned}$$

22b $$\begin{aligned} \varvec{A}_\mathrm {m} \ = \ \varvec{A}_\mathrm {f} \ , \ \text {on} \ \mathrm {\Gamma _m} \setminus \mathrm {\Gamma _b} \ , \end{aligned}$$

22c $$\begin{aligned} \varvec{A}_\mathrm {b} \ = \ \varvec{A}_\mathrm {f} \ , \ \text {on} \ \mathrm {\Gamma _b^{out}} \ . \end{aligned}$$

Further, for biological tissues, surface currents are assumed to be negligible (i.e. they only exhibit volume conduction). Accordingly, the fluxes of the magnetic vector potential across any boundary are balanced Griffiths (2013), i.e.

23a $$\begin{aligned} \big [ {\text {grad}} \, \varvec{A}_\mathrm {b} \, - \, {\text {grad}} \, \varvec{A}_\mathrm {m} \big ]&\cdot \varvec{n}_\mathrm {m} \ = \ \varvec{0} \ , \ \text {on} \ \mathrm {\Gamma _m} \cap \mathrm {\Gamma _b} \ , \end{aligned}$$

23b $$\begin{aligned} \big [ {\text {grad}} \, \varvec{A}_\mathrm {f} \, - \, {\text {grad}} \, \varvec{A}_\mathrm {m} \big ]&\cdot \varvec{n}_\mathrm {m} \ = \ \varvec{0} \ , \ \text {on} \ \mathrm {\Gamma _m} \setminus \mathrm {\Gamma _b} \ , \end{aligned}$$

23c $$\begin{aligned} \big [ {\text {grad}} \, \varvec{A}_\mathrm {f} \, - \, {\text {grad}} \, \varvec{A}_\mathrm {b} \big ]&\cdot \varvec{n}_\mathrm {b}^\mathrm {out} \ = \ \varvec{0} \ , \ \text {on} \ \mathrm {\Gamma _b^{out}} \ . \end{aligned}$$

### In silico experiments

**Table 1:** Summary of model parameters

Parameter	Symbol	Value (slow to fast)	Reference
Longitudinal intracellular conductivity	$\sigma _\mathrm {i}^\mathrm {l}$	8.93 mS cm^−1^	Bryant (1969)
Transversal intracellular conductivity	$\sigma _\mathrm {i}^\mathrm {t}$	0.0 mS cm^−1^	cf. Klotz et al. (2020)
Longitudinal extracellular conductivity	$\sigma _\mathrm {e}^\mathrm {l}$	6.7 mS cm^−1^	Rush et al. (1963)
Transversal extracellular conductivity	$\sigma _\mathrm {e}^\mathrm {t}$	3.35 mS cm^−1^	cf. Klotz et al. (2020)
Fat conductivity	$\sigma _\mathrm {b}$	0.4 mS cm^−1^	Rush et al. (1963)
Membrane capacitance	$C_\mathrm {m}^k$	1 μF cm^−2^	Hodgkin and Huxley (1952)
Surface-to-volume ratio	$A_\mathrm {m}^k$	500 cm^−1^	cf. Klotz et al. (2020)
Motor unit density	$f_\mathrm {r}^k$	Variable	
Magnetic permeability	$\mu _0$		

**Fig. 3:**
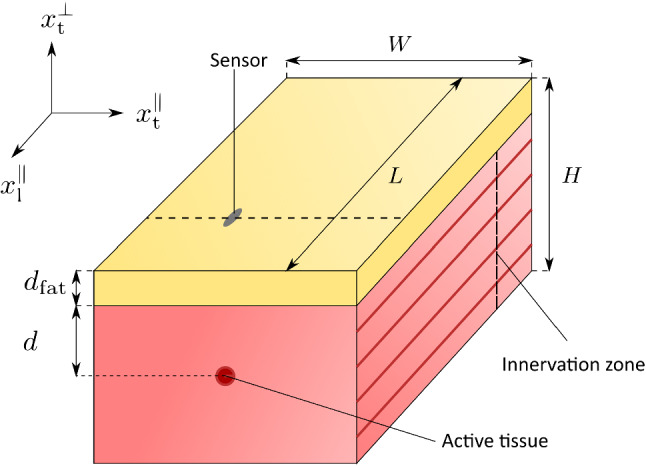
Schematic drawing illustrating the simulated tissue geometry, whereby muscle and fat tissue are coloured in red and yellow, respectively. The muscle fibres are aligned with the $x_\mathrm {l}^{\parallel }$-direction

The main aim of this work is to employ the previously described modelling framework to investigate the spatial selectivity of non-invasive EMG and MMG, i.e. comparing both signals ability to distinguish spatially separated sources. This is achieved by simulating a muscle with a layer of subcutaneous fat on top and which is variable in thickness. We exclude the influence of the geometry by focusing on a cube-shaped (half) muscle sample with edge lengths *L* = 4.0 cm, *W* = 1.5 cm and *H* = 2.0 cm (cf. Fig. 3). The muscle fibres are aligned with the longest edge, i.e. denoted as the $x_\mathrm {l}^{\parallel }$-direction. The spatial selectivity is tested by a set of in silico experiments, whereby the muscle fibres, i.e. the intracellular domains, are selectively stimulated at different depths, i.e. at *d*= 0.3 cm, 0.5 cm, 0.7 cm, 0.9 cm and 1.1 cm. To do so, we first subdivide the muscle into two motor units. All recruited fibres are grouped into the first motor unit (MU1). The territory of MU1 is defined by all points at the cross sectional coordinates $x_\mathrm {t}^{\parallel }=0.75 \,\mathrm{cm}$ and $x_\mathrm {t}^{\perp }=2 \,\mathrm{cm} - d$. The territory of the second motor unit (MU2) contains all points that are not included in the territory of MU1. Hence, for both motor units, we choose $f_\mathrm {r}^k = 1$ ($k=1,2$). To stimulate the fibres, a single current pulse with amplitude 700 mA cm$^{-2}$ and length 0.1 ms is applied to the muscle fibre membranes of MU1 at their neuromuscular junctions, i.e. at $x_\mathrm {l}^{\parallel }=1\,\mathrm{cm}$, $x_\mathrm {t}^{\parallel }=0.75 \,\mathrm{cm}$ and $x_\mathrm {t}^{\perp }=2 \,\mathrm{cm} - d$. In order to study the filtering effect of the subcutaneous tissues, a reference simulation is conducted for an isolated muscle (i.e. $d_\mathrm {fat}=$ 0.0 cm) and compared to the results obtained for two configurations including subcutaneous fat, i.e. for adipose tissue layers with $d_\mathrm {fat}=$ 0.2  cm and $d_\mathrm {fat}=$ 0.4 cm. All other model parameters are summarised in Table 1. Based on these parameters, the intracellular conductivity tensors are calculated by $\varvec{\sigma }_\mathrm {i}^k = \sigma _\mathrm {i}^\mathrm {l} \, \varvec{f} \otimes \varvec{f}$ ($\forall k \in \mathscr {M}_\mathrm {MU}$), where $\varvec{f}$ is a unit vector aligned with the muscle fibre direction. Accordingly, the extracellular conductivity tensor is given by $\varvec{\sigma }_\mathrm {e} = \sigma _\mathrm {e}^\mathrm {l} \, \varvec{f} \otimes \varvec{f} \, + \, \sigma _\mathrm {e}^\mathrm {t} \, \left( \varvec{I} - \varvec{f} \otimes \varvec{f} \right)$ with $\varvec{I}$ being the second-order identity tensor. To simulate the behaviour of the muscle fibre membranes we appeal to the model of Hodgkin and Huxley (1952), which was imported from the models repository of the Physiome Project^1^ (cf. Lloyd et al. (2004)). Including a sodium and a potassium conductance, this can be considered as a basis model for the electric behaviour of the muscle fibre membranes. Finally we note that the given model can only be solved numerically and the applied methods are presented in Appendix.

### Virtual EMG and MMG recordings and data analysis

The computational model yields at each time step and each grid point a prediction for the electric potential in each domain and for the magnetic field. We assume an idealised recording system that does not affect the physical fields. It measures at a selected discrete location (i.e. channel) the extracellular potential (or the body potential) and all three components of the magnetic field yielding a measurement vector

24 $$\begin{aligned} \varvec{m}(\varvec{x},t) = {\left\{ \begin{array}{ll} {[}\phi _\mathrm {e}, B_\mathrm {l}^{\parallel }, B_\mathrm {t}^{\parallel }, B_\mathrm {t}^{\perp }]^T &{} \varvec{x} \in {\Omega }_\mathrm {m} \\ {[}\phi _\mathrm {b}, B_\mathrm {l}^{\parallel }, B_\mathrm {t}^{\parallel }, B_\mathrm {t}^{\perp }]^T &{} \varvec{x} \in {\Omega }_\mathrm {b} . \end{array}\right. } \end{aligned}$$

Therein $B_\mathrm {l}^{\parallel }$ is the magnetic field component aligned with the muscle fibres (and tangential to the muscle surface), $B_\mathrm {t}^{\parallel }$ is the component of the magnetic field orthogonal to the muscle fibres and tangential to the surface, and $B_\mathrm {t}^{\perp }$ is the magnetic field component normal to the body surface (and orthogonal to the muscle fibres), cf. Fig. 3. We assume a sampling frequency of 10,000 Hz for both the synthetic EMG and MMG.

The decay of the amplitude when increasing the distance between source and sensor is an important feature for evaluating the spatial selectivity of a recording system. Hence, for each numerical experiment the root-mean-square (RMS) value is calculated for the virtual EMG and MMG signals. Further, a modulation in a signal’s frequency content provides insights if a bioelectric source is distorted by the intrinsic tissue properties. The spectral content of the virtual signals is investigated by estimating the power spectral density (PSD).

## Results

### Single channel recordings at the muscle surface

**Fig. 4:**
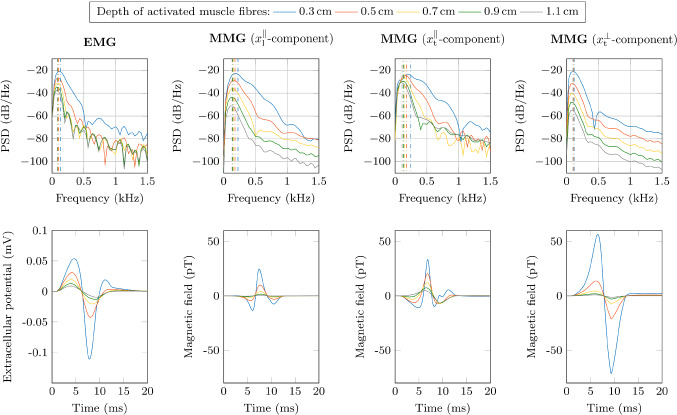
Power spectral density (PSD) and time domain graph of the simulated surface EMG signal and surface MMG signal for variable depths of the activated muscle tissue (blue: 0.3 cm; red: 0.5 cm; yellow: 0.7 cm; green: 0.9 cm; grey: 1.1 cm). The virtual sensor is placed at an arbitrary chosen virtual recording point ($x_1 = 3 \,\mathrm{cm}$, $x_2 = 0.6 \,\mathrm{cm}$). Each spectrum is normalised by the total power of the respective EMG/MMG signal obtained from the simulation with a depth of 0.3 cm. The dashed lines in the power spectrum (top row) indicate the mean frequency content of the signal

As baseline experiment, the spatial resolution of EMG and MMG signals is investigated for an isolated muscle. To do so, the muscle fibres are selectively stimulated in different depths within the muscle tissue (cf. Sect. 2.2). The muscle response is observed from a single channel, which is placed between the innervation zone and the boundary of the muscle on its surface (cf. Fig. 3), i.e. $x_\mathrm {l}^{\parallel } = 2.5\,\mathrm{cm}$ and $x_\mathrm {t}^{\parallel } = 0.6\,\mathrm{cm}$. The bottom row of Fig. 4 shows that the amplitude of all components of measurement vector $\varvec{m}$ (i.e. the extracellular potential $\phi _\mathrm {e}$ and three components of the magnetic field $\varvec{B}$) decreases with increasing activation depth. In detail, the decrease in amplitude is most distinct for the surface normal component of the magnetic field, i.e. for a depth of 1.1 cm the RMS decreases by a factor of 0.019 if compared to the RMS at $d=$ 0.3 cm (cf. Table 2). The signal decay is least pronounced for the magnetic field component tangential to the body surface and orthogonal to the muscle fibre direction, i.e. for a depth of 1.1 cm the RMS decreases by a factor of 0.317 of the RMS at *d*= 0.3 cm. For the same condition the RMS of the EMG decreases by a factor of 0.124 and the MMG component aligned with the muscle fibres decreases by a factor of 0.031. Further, from Fig. 4 and Table 2 it can be seen that increasing the depth of the stimulated fibres causes a left-shift in the mean frequency content of the observed signals. While this observation is partially explained by the spatio-temporal properties of the sources, i.e. the propagating nature of the action potentials, a strong compression of the frequency content indicates a low-pass filtering effect of the muscle tissue. Here, the surface normal component of the magnetic field exhibits the lowest frequency modulation. Further, the shift in the mean frequency content is relatively smaller for the EMG than for the $x_\mathrm {l}^{\parallel }$-component and the $x_\mathrm {t}^{\parallel }$-component of the MMG.

**Table 2:** Effect of the depth of the activated muscle fibres on the RMS and the MNF of the surface EMG signal and surface MMG signal. Note that for the virtual MMG recordings each component of the magnetic field is measured individually and which is indicated by the respective coordinate shown in brackets. All values are normalised with respect to the values from the simulation with the lowest depth, i.e. 0.3 cm

Depth (cm)	0.3	0.5	0.7	0.9	1.1
EMG-RMS	1	0.468	0.270	0.177	0.124
EMG-MNF	1	0.802	0.714	0.678	0.660
MMG-RMS ($x_\mathrm {l}^{\parallel }$)	1	0.445	0.184	0.076	0.031
MMG-MNF ($x_\mathrm {l}^{\parallel }$)	1	0.788	0.699	0.639	0.592
MMG-RMS ($x_\mathrm {t}^{\parallel }$)	1	0.728	0.507	0.3766	0.3171
MMG-MNF ($x_\mathrm {t}^{\parallel }$)	1	0.715	0.598	0.521	0.444
MMG-RMS ($x_\mathrm {t}^{\perp }$)	1	0.286	0.101	0.041	0.019
MMG-MNF ($x_\mathrm {t}^{\perp }$)	1	0.902	0.858	0.847	0.854

### Single channel recordings at the body surface

**Fig. 5:**
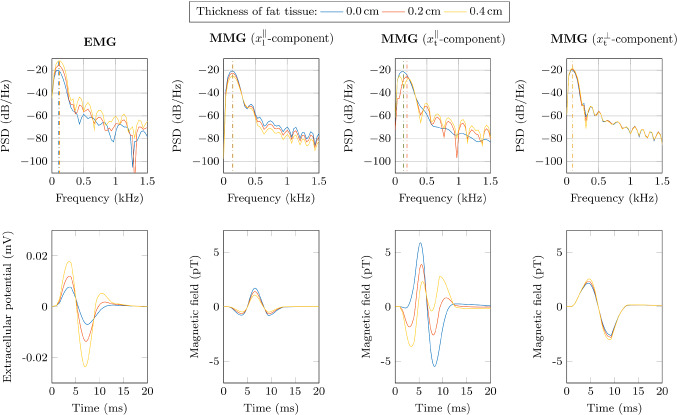
Power spectral density (PSD) and time domain graph of the simulated surface EMG and surface MMG signal for variable thicknesses of a superficial adipose tissue layer (blue: 0.0 cm; red: 0.2 cm; yellow: 0.4 cm). The distance between the active fibres and the sensor location ($x_\mathrm {l}^{\parallel } = 2.5\, cm$, $x_\mathrm {t}^{\parallel } = 0.6\, \mathrm{cm}$) is fixed. Each spectrum is normalised by the total power of the respective EMG/MMG signal obtained from the simulation with no fat. The dashed lines in the power spectrum (top row) indicate the mean frequency content of the signal

To investigate the influence of adipose tissue on non-invasively observable surface signals, we compare the computed fields for three cases with variable fat tissue thickness, i.e. 0 cm, 0.2 cm and 0.4 cm. The distance between the recording point and the active fibres is kept constant. Hence, when a thicker fat tissue layer is simulated more superficial fibres are stimulated, i.e. $d=$ 0.9 cm, 0.7 cm and 0.5 cm, respectively. Again, the muscle’s response is observed from a single channel at $x_\mathrm {l}^{\parallel } = 2.5 \,\mathrm{cm}$, and $x_\mathrm {t}^{\parallel } = 0.6\, \mathrm{cm}$. Figure 5 depicts that the amplitude of the surface signal strongly depends on the thickness of the fat tissue layer for the EMG. The same holds for the $x_\mathrm {t}^{\parallel }$-component and the $x_\mathrm {f}^{\parallel }$-component of the MMG. In detail, for the in silico experiments with fat tissue layers of 0.2 cm and 0.4 cm, the RMS of the EMG signal increases by a factor of 1.67 and 2.72 when compared to the case without fat. For the MMG component aligned with the muscle fibres, the RMS decreases by a factor of 0.80 and 0.61, respectively. As far as the $x_\mathrm {t}^{\parallel }$-component of the MMG is concerned, the RMS values change by a factor of 0.55 ($d_\mathrm {fat}=$ 0.2 cm) and 0.63 ($d_\mathrm {fat}=$ 0.4 cm) compared to the respective reference RMS value without fat. Thereby, one also observes a notably modulated shape of the surface potential. This is also reflected by a change of the signal’s frequency spectrum. In contrast, the amplitude and the frequency content of the normal-to-the-body-surface component are less affected by the adipose tissue. For the in silico experiment with $d_\mathrm {fat}=$ 0.4 cm, the RMS value of the $x_\mathrm {t}^{\perp }$-component changes only by a factor of 1.16 compared to the simulation without fat.

### The signal amplitude on multiple surface channels

**Fig. 6:**
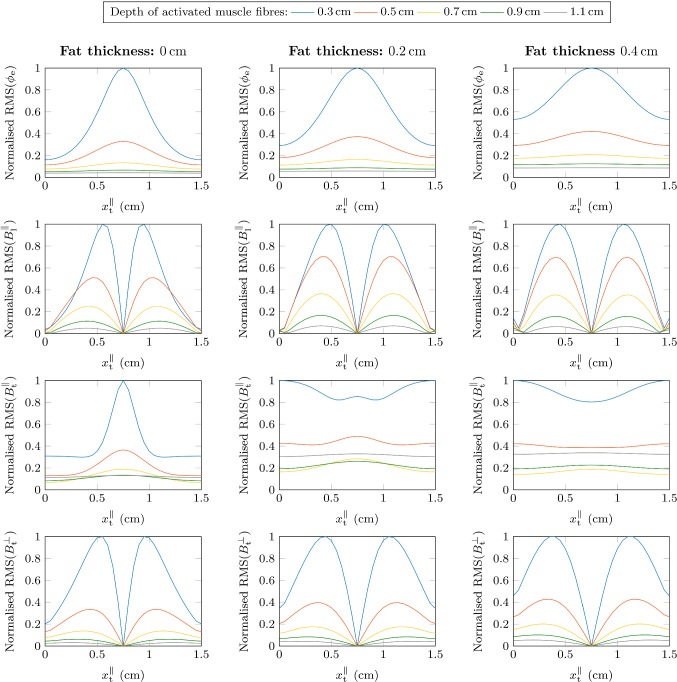
Normalised RMS values for differently thick fat tissue layers. The measurements are in a line orthogonal to the muscle fibres between the innervation zone and the muscle boundary, i.e. $x_\mathrm {l}^{\parallel } = 2.5 \,\mathrm{cm}$ on the surface of the tissue sample. From left to right the thickness of the fat tissue increases. The first row shows the EMG, the second row shows the $x_\mathrm {l}^{\parallel }$-component of the MMG, the third row shows the $x_\mathrm {t}^{\parallel }$-component of the MMG and the forth row shows $x_\mathrm {t}^{\perp }$-component of the MMG. Further, the colours indicate the depth of the activated muscle fibres (blue: 0.3 cm; red: 0.5 cm; yellow: 0.7 cm; green: 0.9 cm; grey: 1.1 cm)

Further insights on the spatial selectivity of both EMG and MMG signals can be gained, when considering the dependency between the sensor position and the spatio-temporal properties of the bioelectric sources. To do so, we evaluate the root mean square (RMS) for all components of the measurement vector $\varvec{m}$ in a line orthogonal to the muscle fibres and mid way through the innervation zone and the muscle boundary (cf. Fig. 3). Figure 6 shows that the spatial distribution of the signal’s power is fundamentally different between the EMG and the MMG-components. For the EMG signal, the amplitude reaches its maximal value directly over the active fibres. For the $x_\mathrm {l}^{\parallel }$-component and $x_\mathrm {t}^{\perp }$-component of the MMG, the signal’s amplitude is zero directly over the source. Further, the depth of the active fibre correlates with the distance to the maximum. Considering the case without fat, the distance between the zero value of the $x_\mathrm {l}^{\parallel }$-component (directly over the source) and the maximal RMS value is 0.2 cm for a fibre depth of 0.3 cm, 0.3 cm for a fibre depth of 0.5 cm, and saturates at 0.35 cm for higher fibre depths. Similarly, for the $x_\mathrm {t}^{\perp }$-component and in the case without fat, the distance between the maximum RMS value and the zero value is 0.2 cm for a fibre depth of 0.3 cm, 0.3 cm for a fibre depth of 0.5 cm, 0.4 cm for a fibre depth of 0.7 cm, 0.45 cm for a fibre depth of 0.9 cm and 0.5 cm for a fibre depth of 1.1 cm. Further, it can be seen that the RMS distribution of the $x_\mathrm {t}^{\parallel }$-MMG-component strongly depends on the fat tissue layer and does not follow a distinct pattern. When increasing the thickness of the fat tissue layer, for the EMG it can be observed that the inter-channel variability gets strongly compressed. For example, for a fibre depth of 0.3 cm the coefficient of variation of the RMS values is 63.6% for the case without fat, 43.4% for $d_\mathrm {fat}=$ 0.2 cm and 22.9% for $d_\mathrm {fat}=$ 0.4 cm. In contrast, the MMG components aligned with the muscle fibres and normal to the surface better preserve the inter-channel variability. Considering the in silico experiment with a fibre depth of 0.3 cm, the coefficient of variation of the RMS values for the $x_\mathrm {l}^{\parallel }$-component is 73.1% in the case there is no fat, 61.6% for $d_\mathrm {fat}=$ 0.2 cm and 58.6% for $d_\mathrm {fat}=$ 0.4 cm. For the $x_\mathrm {t}^{\perp }$-component the coefficient of variation of the RMS values is 49.5% in the case without fat, 40.2% for $d_\mathrm {fat}=$ 0.2 cm and 37.9% for $d_\mathrm {fat}=$ 0.4 cm.

### The contribution of different domains to the magnetic field

**Fig. 7:**
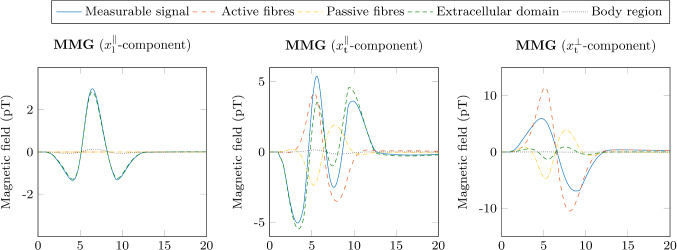
Contribution of different domains to the (magnetic) muscle action potential when selectively stimulating muscle fibres at $x_\mathrm {l}^{\parallel } = 1\, \mathrm{cm}$, $x_\mathrm {t}^{\parallel }= 0.75\, \mathrm{cm}$ and $x_\mathrm {l}^{\perp } = 1.5\, \mathrm{cm}$. The virtual sensor is placed at an arbitrary point on the muscle surface ($x_\mathrm {l}^\mathrm {\parallel } = 2.5\, \mathrm{cm}$, $x_\mathrm {t}^{\parallel } = 0.6\, \mathrm{cm}$)

To investigate the origin of the experimentally observable magnetic fields, the MMG recorded on the body surface is split up into the contribution of the different domains. To do so, we exploit the linearity of the magnetic field equations, i.e. Eqs. 13 and 14. Therefore, the solution of the overall magnetic field problem can be reconstructed by adding up the individual solutions of each right hand term, i.e. the contribution of each domain/region (cf. Sect. 2.1.4). In Fig. 7 this is exemplary shown for the in silico experiment with a fat tissue layer of 0.2 cm and active muscle fibres in a depth of 0.5 cm. It can be observed that the component of the magnetic field aligned with the muscle fibres, i.e. the $x_\mathrm {l}^{\parallel }$-component, is completely determined by volume currents in the extracellular space and the body region. The RMS of the extracellular contribution is 0.950 and the RMS of the body region contribution is 0.051 (normalised with respect to the RMS value of the observable magnetic field). In contrast, the magnetic field components orthogonal to the muscle fibre direction, i.e. the $x_\mathrm {t}^{\parallel }$-component and the $x_\mathrm {t}^{\perp }$-component, depend on currents from all domains. Thereby, the non-recruited muscle fibres considerably contribute to the experimentally observable magnetic field; for the presented simulation, the currents in the active and passive muscle fibres have opposite directions and thus mutually limit their visibility in the observable magnetic field. In detail, for the $x_\mathrm {t}^{\parallel }$-component the domain specific RMS values normalised with respect to the measurable field are 0.999 for the extracellular space, 0.670 for the active intracellular domains, 0.357 for the non stimulated intracellular domains and 0.027 for the body region. Considering the normal-to-the-body-surface component, then the active fibres dominate the measurable signal, i.e. the RMS normalised with respect to the RMS value of the observable magnetic field is 1.485. Further, the normalised RMS values are 0.517 for the passive intracellular domains, 0.134 for the extracellular space and 0.002 for the body region.

## Discussion

Within this work we propose a novel in silico framework to simulate electro-magnetic fields induced by the activity of skeletal muscles. The model is used for the first systematic comparison between the well-established EMG measurements, cf. Merletti and Farina (2016), and MMG which recently gained attention due to progress in sensor technology (cf. e.g., Zuo et al. (2020); Broser et al. (2018, 2021); Llinás et al. (2020)).

### Limitations

A direct validation of the proposed simulation framework is currently out of scope due to methodological limitations. However, as the proposed model consistently integrates a validated microscopic electric circuit model of the muscle fibre membranes, i. e., the biophysical origin of bioelectromagnetic fields from skeletal muscles, into a macroscopic continuum field model solving Maxwell’s equations, we strongly believe that the proposed multi-scale model has strong predictive capabilities. This is underlined by the fact that the proposed simulation framework respects various experimental observations. In Klotz et al. (2020) we have already shown that the multi-domain modelling framework is capable of replicating the key characteristics (e.g., the shape, amplitude, conduction velocity and frequency content of motor unit action potentials) of experimentally observable EMG signals. The same amount, type and quality of data are not available for MMG. Nevertheless, the proposed simulation framework is qualitatively in agreement with available experimental observations. First, the model respects the observation that motor unit action potentials recorded with EMG and MMG have the same temporal duration as well as showing similar frequency content Cohen and Givler (1972), Parker and Wikswo (1997) and Zuo et al. (2020). Further, the biphasic shape of the contribution of the active muscle fibres to the total magnetic field (cf. Fig. 7) is perfectly in agreement with the measurement of the magnetic field of an isolated muscle fibre presented in Egeraat et al. (1990). As experimentally measured by Broser et al. (2021), the proposed model predicts a triphasic shape of the overall magnetic muscle action potential caused by volume currents in the extracellular space. However, as volume currents strongly depend on the exact muscle geometry, a direct comparison to the experiments of Broser et al. (2021) is not possible. Nevertheless, it should be noted that the presented continuum field approach provides a high flexibility to resolve arbitrary muscle geometries by employing discretization schemes such as the finite element method, e.g., Heidlauf et al. (2016), Mordhorst et al. (2015) and Schmid et al. (2019). We further note that within this work we focused on the physical properties of the bioelectromagnetic fields. Hence, we considered idealised sensors that can record from a single point in space and measurements are unaffected by noise. However, for comparing the model predictions with a specific experiment the sensor properties need to be considered.

### The spatial resolution of EMG and MMG

The spatial selectivity is one of the most extensively discussed properties of EMG. That is, when employing invasive needle electrodes, EMG is highly sensitive to the location of the measurement. However, when recorded non-invasively from the skin EMG’s spatial selectivity is strongly compressed as surrounding electrically inactive tissues, such as, for example, fat, act as a low-pass filter. Within this work we address the hypothesis that non-invasive MMG can overcome the limitations of surface EMG’s spatial selectivity. We did so by carrying out an in silico comparison between both bioelectric and biomagnetic signals.

EMG and MMG measure different physical fields and therefore are not directly comparable. Thus, as a reference experiment we investigated the spatial selectivity of EMG and MMG signals directly recorded from the surface of an isolated muscle. When the distance between the recording point and the active muscle fibres is increased, all MMG components and the EMG show a strong decrease in amplitude. Hence, we conclude that when directly observed from the muscle surface both electric potential and magnetic field recordings should show a reasonable spatial selectivity to separate spatially distinct sources. This, however, changes, if we consider non-invasive surface recordings (which are affected by electrically inactive tissues such as fat). Our simulations show, as previously reported, e.g. Roeleveld et al. (1997), Lowery et al. (2002) and Farina et al. (2002), that the spatial selectivity of the EMG is compromised. We conclude this from the fact that increasing the thickness of the adipose tissue causes a strong modulation of the EMG signal’s amplitude (cf. Figs. 5 and 6).

Considering the MMG’s $x_\mathrm {t}^{\parallel }$-component, the effect of fat tissue on the surface signal is even more pronounced than for EMG. However, in comparison with EMG, our simulations show that the MMG components normal to the surface and aligned with the muscle fibres exhibit a much less pronounced influence of subcutaneous tissues. Particularly, the spatio-temporal pattern of the normal-to-the-surface component is nearly preserved (cf. Figs. 5 and 6). Thus, we conclude that a careful selection of the measured magnetic field component can overcome the limitations given by the poor spatial selectivity of surface EMG.

The potentially most interesting implication of surface MMG’s higher spatial selectivity is the increased variability of the motor unit action potentials observable from the body surface. This advocates for the use of non-invasive MMG recordings to decode the neural drive to a muscle using source separation techniques, e.g. Nawab et al. (2010), Holobar et al. (2010), Farina et al. (2014) and Negro 2016, as the close similarity of multiple motor unit action potentials is one major limitation when decomposing high-density EMG signals. Further, one can speculate that the consistently fast decay of the MMG’s amplitude limits the signal’s contamination with cross-talk—another well-documented limitation of surface EMG. On the other hand, it should be noted that a higher spatial selectivity also implies that rather local properties of the muscle tissue are observed. This, if not compensated by a congruous amount of sensors, may compromise the robustness and comparability of measurements as a too pronounced weighting of local properties yields the risk to bias the observations. This is a well-known limitation of intramuscular EMG decomposition (cf., e.g. De Luca et al. 2006; Farina et al. 2010; Farina and Negro 2012). Further, it is noted that a higher spatial selectivity makes measurements more susceptible for motion artefacts.

### The biophysical origin of the measurable magnetic field

We make use of the systemic modelling framework to deduce the biophysical origin of the magnetic field induced by muscle activity. An electric current only can generate a magnetic field circular to the direction of the current. Accordingly, we showed that the magnetic field aligned with the muscle fibres, is fully determined by volume currents in the extracellular space/surrounding tissues. In contrast, both magnetic field components orthogonal to the muscle fibre direction contain contributions from intracellular currents, which are in the literature sometimes referred to as primary currents Malmivuo and Plonsey (1995). However, while the MMG component tangential to the body surface and orthogonal to the muscle fibres, i.e. the $x_\mathrm {t}^{\parallel }$-component, is dominated by volume currents, the surface-normal component of the MMG, i.e. the $x_\mathrm {t}^{\perp }$-component, is dominated by intracellular currents.

The observation that the surface-normal component of the magnetic field strongly reflects intracellular currents and is relatively insensitive to the effect of fat, yields several potential benefits for the interpretation of experimental data. This can be beneficial when properties on the muscle fibre level, for example, membrane fatigue, should be estimated from MMG data. Further, when aiming to use inverse modelling and MMG to reconstruct the sources of the bioelectromagnetic activity, e.g. Llinás et al. (2020), a field component which is (nearly) invariant with respect to volume currents can reduce the uncertainty associated with the required estimate for the tissue’s conductive properties. We conclude this discussion by noting that the model predicts a surprisingly big contribution of passive muscle fibres.

## Conclusion and outlook

Within this work we propose a systemic multi-scale model to simulate EMG and MMG. We show that non-invasive MMG can overcome the limitations of surface EMG, in particular with respect to its poor spatial selectivity. In the future, we want to use the presented modelling framework to investigate the potential of non-invasive MMG to study voluntary contractions. Particularly the potential to improve the accuracy of state-of-the-art motor unit decomposition methodologies seems to be attractive. Further, given the emerging progress in MMG sensor technology, the presented systemic simulation framework provides excellent capabilities to assist the interpretation of experimental data as well as assisting the optimisation of MMG sensor arrays.

## Numerical treatment

The mathematical modelling framework presented in this work, i.e. Sect. 2.1, can only be solved numerically. The applied methods are outlined in the following. In summary, we exploit the quasi-static conditions and decouple the electric and the magnetic field (cf. Sect. 2.1.1). Thus, we appeal to a staggered solution scheme where (i) the electric field equations are solved for the muscle as well as the body domain and (ii) the magnetic field predictions are based on the previously calculated electric potentials. Therefore, the continuous domains are represented by a finite number of grid points and the spatial derivatives are approximated by finite differences. The whole model is implemented in MATLAB (The MathWorks, Inc., Natick, Massachusetts, USA) and the corresponding code is hosted on a freely accessible git repository^2^.

### Solving for the electrical potential fields

Given the reaction-diffusion characteristic of Eq. (10b), a first-order Godunov-type splitting scheme is applied to yield

25a $$\begin{aligned} \frac{V_\mathrm {m}^{k,t_*}-V_\mathrm {m}^{k,t_i}}{\varDelta t} \ = \ - \frac{1}{C_\mathrm {m}^k} \, I_\mathrm {ion}^k(\varvec{y}^k,V_\mathrm {m}^k,I_\mathrm{stim}^k) \ , \end{aligned}$$

25b $$\begin{aligned} \frac{{V_{{\text{m}}}^{{k,t_{{i + 1}} }} - V_{{\text{m}}}^{{k,t_{*} }} }}{\varDelta t} \ & = \;\frac{1}{{C_{{\text{m}}}^{k} A_{{\text{m}}}^{k} }}{\mkern 1mu} \big({\text{div}}\left[ {\boldsymbol{\sigma}_{{\text{i}}}^{k} {\text{grad}} \, V_{{\text{m}}}^{k} } \right]{\mkern 1mu} \\ & \quad +\, {\text{div}}\left[ {\boldsymbol{\sigma}_{{\text{i}}}^{k} {\text{grad}} \, \phi _{{\text{e}}} } \right]\big)\;, \\ \end{aligned}$$

for each skeletal muscle material point $\mathcal {P}\in \mathrm {\Omega _m}$ and $k \in \mathscr {M}_\mathrm {MU}$. Therein, a first-order forward finite difference is used to approximate the temporal derivatives and $t_*$ denotes an intermediate time step. Note that the hereby introduced splitting error becomes acceptable for sufficiently small time steps. Herein, we chose as time step $\varDelta t = 0.1 \, \text{ms}$. Further, note that both Eqs. (25a) and (25b) are still continuous in space. This allows us to use specialised solution schemes for the reactive and the diffusive parts of the model.

In detail, Eq. (25a) together with Eq. (10c) forms a system of stiff ordinary differential equations, which is solved for the interval $[t_i, t_*]$ by an improved Euler method and a fixed time step of $\varDelta t_\mathrm {ode} = 0.001 \, \text{ms}$ (cf. Bradley et al. (2018)). Further, the coupled diffusion problem given by given by Eq. (10a), Eq. 25b and Eq. (11), is addressed by evaluating Eqs. (10a), (11) and the right-hand side of Eq. (25b) at $t=t_{i+1}$, i.e. employing an implicit Euler method, whereby the spatial derivatives are approximated with second-order accurate central finite differences. Accordingly, the flux boundary conditions, i. e., Eqs. (16), (17), (18) and (20), are also evaluated at $t=t_{i+1}$, while being approximated with second-order accurate forward/backward finite differences. For the spatial discretisation, we chose equally spaced grid points and a step size of $h=0.05\, {\rm cm}$. This discretisation yields a linear system of equations which is solved with MATLAB’s built-in GMRES function Saad and Schultz (1986). The linear system is preconditioned via an incomplete LU factorisation (Crout version, drop tolerance: 1e-6) and the following solver options are applied: an absolute and relative tolerance of 1e-10, restart after 20 inner iterations and a maximum number of 20 outer iterations.

### Solving for the magnetic vector potential

Based on the solution of the multi-domain model, i.e. the electric potential fields for each time step and each grid point of the muscle region and body region, second-order central finite differences are used to obtain estimates for the (first) spatial derivative of the electrical potentials of the right-hand side of Eqs. (13) and (14). Further, the (second) spatial derivatives given on the left-hand side of Eqs. (13) and (14) are discretised using second-order central finite differences.

As for the electric potential fields, the flux boundary conditions of the magnetic vector potential in the muscle and body region (cf. Eq. (23)) are approximated with second-order accurate forward/backward finite differences. The surrounding air is represented by an infinitely long (virtual) boundary element. Its normal derivative (cf. Eq. (23)) is approximated by a first-order forward/backward finite difference. Recalling that the magnetic vector potential is zero far away from the bioelectric sources (cf. Eq. (21)), the normal derivative of the magnetic vector potential vanishes at the interface between the body and air.

In summary, this discretisation yields a linear system of equations, which needs to be solved to obtain the magnetic vector potential at each grid point of the muscle and the body region. This linear system is solved with MATLAB’s build in ”mldivide” function, as this function can handle multiple pre-computed right-hand side vectors simultaneously. Finally, the magnetic field $\varvec{B}$ is calculated via Eq. (3) in a post-processing step. Thereby the (first) spatial derivatives of the curl operator are approximated by second-order central finite differences.

## Footnotes

1 https://models.physiomeproject.org/cellml.
